# DNA methylation in normal-appearing tissue associated with prostate cancer recurrence and metastasis

**DOI:** 10.1186/s13148-025-01932-x

**Published:** 2025-07-21

**Authors:** Christine Aaserød Pedersen, Thomas Fleischer, Maximilian Wess, Elise Midtbust, Maria K. Andersen, Trond Viset, Øystein Størkersen, Morten B. Rye, May-Britt Tessem

**Affiliations:** 1 Department of Circulation and Medical Imaging, NTNU, Trondheim, Norway; 2 Department of Cancer Genetics, Institute for Cancer Research, Oslo University Hospital, Oslo, Norway; 3 Clinic of Surgery, St. Olavs Hospital, Trondheim University Hospital, Trondheim, Norway; 4 Department of Pathology, St. Olav Hospital, Trondheim University Hospital, Trondheim, Norway; 5 Department of Clinical and Molecular Medicine, NTNU, Trondheim, Norway; 6 Department of Clinical and Molecular Medicine, NTNU – Norwegian University of Science and Technology, Trondheim, Norway; 7 Clinic of Laboratory Medicine, St. Olavs Hospital, Trondheim University Hospital, Trondheim, Norway; 8 BioCore - Bioinformatics Core Facility, NTNU – Norwegian University of Science and Technology, Trondheim, Norway

## Abstract

There is a need for more precise biomarkers and understanding on the development of aggressive prostate cancer. In this study, we analyzed DNA methylation in 64 prostate cancer tissue samples, using tissue from radical prostatectomy patients (*n* = 16) with up to 16 years of clinical follow-up. We used several samples from each patient including both normal and cancer tissue to study DNA methylation patterns in relation to aggressiveness measured by follow-up data of biochemical recurrence and metastasis status as clinical endpoints. We identified differentially methylated CpGs associated with recurrence and metastasis, regardless of whether the tissue was normal, cancer-adjacent normal, or cancer. The identified CpG sites were over-represented in promoter regions and transcription factor binding regions, suggesting their influence on gene expression regulation. They further exhibited low intrapatient heterogeneity both between normal, normal adjacent, and cancer tissue, making them favorable as potential biomarkers for aggressive prostate cancer. However, validation of a subset of these CpGs in an external dataset was unsuccessful.

**Supplementary Information:**

The online version contains supplementary material available at 10.1186/s13148-025-01932-x.

## Introduction

Prostate cancer ranks among the most prevalent cancers in men globally, with an increased incidence due to an aging population. Early-stage diagnosis poses a challenge in distinguishing between patients with slow-growing, indolent prostate cancer and those with aggressive tumors at high risk for metastasis and cancer-specific mortality. There is a significant need for tools to perform more precise patient stratification. This would help minimize unnecessary treatment side effects in patients with indolent cases, while ensuring adequate treatment for those with aggressive disease.

Prostate cancer is known for its high tissue heterogeneity and its multifocality, meaning that one patient can have several separate tumors within the same organ [1]. Different lesions in the same prostate may have different tumor grades and genomic aberrations, and each lesion may also have different areas with different grades and molecular aberrations [2]. This heterogeneity poses a challenge for biomarker discovery and development of clinical biomarkers. Since not all tissue areas exhibit the same molecular profile, clinical decisions can vary based on the specific tumor location that biopsies were sampled from. It is, therefore, important to develop biomarkers that either take this heterogeneity into account, or that remain consistent across the whole prostate.

DNA methylation aberrations are molecular changes affecting the regulation of gene expression without altering the genetic code. DNA methylation is an epigenetic mechanism that involves the addition of methyl groups (CH3) to DNA. This process influences both the binding of transcription factors to DNA and regulates chromatin structure by affecting nucleosome binding. It is hypothesized to be one of the earliest molecular aberrations in cancer development [3], and DNA methylation changes have not only been found in cancerous tissue, but in tissue close to cancer [4–6] and in precancerous lesions [7]. Cancer-related methylation in normal tissue adjacent to the tumor, known as the "field effect," has an unknown mechanism of origin, whether it is a precursor to cancer or a consequence of tumor development. Nonetheless, it holds clinical promise, as methylation patterns in histopathologically normal tissue can be used to diagnose cancer [8, 9].

DNA methylation biomarkers have been proposed to aid in diagnosis and prognostication of prostate cancer patients, even using normal prostate tissue in false-negative biopsies [10]. However, high molecular heterogeneity in the cancer tissue has made it difficult for any of these proposed biomarkers to reach widespread clinical use [11]. In addition, methylation associated with recurrence and aggressive disease compared to slow-growing disease has shown more subtle differences in methylation than between cancer and benign tissue [12]. To improve our biological understanding of how DNA methylation may impact prostate cancer, we assessed widespread methylation from several samples from multiple tissue types per patient using both recurrence and metastasis status as clinical endpoints. In addition, we investigated how the methylation patterns were related to matching gene expression, CpG islands, and chromatin states. This allowed for investigating new biomarkers for prostate cancer aggressiveness independent of tissue heterogeneity and sampling location.

## Methods

### Patient inclusion and characteristics

We collected 64 samples (*n* = 35 cancer, *n* = 14 normal adjacent, and *n* = 15 normal distant) from 16 patients (5 non-recurrence and 11 recurrence). All 16 patients underwent radical prostatectomy as a curative treatment for prostate cancer between 2008 and 2015. A 2 mm thick tissue slice was cut from the middle of the prostate immediately after surgical removal, snap-frozen and stored at − 80 °C by personnel at Biobank1®, the research biobank of Central Norway, following the protocol established by Bertilsson et al. [13]. Written informed consent was collected from all patients, and the study was approved by the Norwegian Regional Ethics committee (REC 2017/576) and followed EU and Norwegian ethical regulations.

The clinical follow-up data were collected in a range of 8–16 years after radical prostatectomy and are summarized in Table 1. Recurrence after surgery was defined by biochemical recurrence (Prostate-specific antigen (PSA) above 0.2 ng/ml after radical prostatectomy). The median follow-up time was 9.2 years (IQR 8.25–12.3 years). The median recurrence-free interval was 3 years (IQR 3.5 months—3.7 years). Metastasis patients (*n* = 5) were diagnosed with metastatic disease using clinical imaging during follow-up.

**Table 1:** Patient characteristics of this study

	Non-recurrence (*N* = 5)	Recurrence (*N* = ll)	Total (*N* = 16)
*Age*			
Median	59.0	63.0	63.0
IQR	56.0, 63.0	61.5, 67.0	59.0, 66.0
*PSA at diagnosis*			
Median	10.2	10.0	10.1
IQR	9.6, 11.5	8.1, 11.5	8.2, 11.5
*EAU risk, n (%)*			
High	2 (40.0%)	3 (27.3%)	5 (31.2%)
Intermediate	3 (60.0%)	8 (72.7%)	11 (68.8%)
*Post-surgery ISUP grade, n (%)*			
2	4 (80.0%)	4 (36.4%)	8 (50.0%)
3	1 (20.0%)	5 (45.5%)	6 (37.5%)
5	0 (0.0%)	2 (18.2%)	2 (12.5%)
*pT stage, n (%)*			
Missing	1	0	1
T2c	4 (100.0%)	4 (36.4%)	8 (53.3%)
T3a	0 (0.0%)	3 (27.3%)	3 (20.0%)
T3b	0 (0.0%)	4 (36.4%)	4 (26.7%)
*Recurrence-free interval (months)*			
Median	–	37.0	37.0
IQR	–	3.5, 44.5	3.5, 44.5
*Metastasis status*			
No metastasis	5 (100.0%)	6 (54.5%)	11 (68.8%)
Metastasis	0 (0.0%)	5 (45.5%)	5 (31.2%)

IQR – interquartile range, EAU – European Association of Urology, and ISUP – International Society of Pathology

### Sample selection and handling

**Fig. 1:**
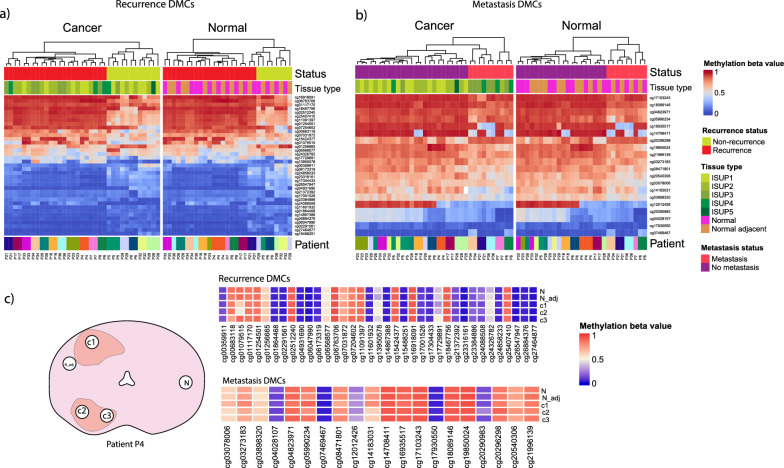
Differentially methylated CpGs (DMCs) associated with recurrence and metastasis (**a**) DMCs according to recurrence status including all sample classes of cancer, normal, and normal adjacent tissue. **b** The metastasis DMCs separated according to metastatic status including all sample classes of cancer, normal, and normal adjacent tissue. **c** An example of a prostate slice (left panel) and methylation levels (right panels) of one of the patients. Heatmaps showing that methylation level of recurrence DMCs and metastasis DMCs within one patient is similar across samples from several locations in the prostate, even across two separate tumors. C1, c2, c3 = cancer samples 1, 2, and 3, N_adj = normal adjacent, and N = normal distant

As part of the clinical routine, the whole prostate is sectioned, paraffin-embedded, and HES-stained for pathology evaluation. The annotation of the HES stain collected closest to the biobanked fresh frozen tissue slice, was used to identify tissue regions of interest and where 3–5 circular samples (3 min in diameter) were drilled out for this study (example shown in Fig. 1c). Each sample was further sectioned and HES-stained to confirm the content in the targeted regions and to accurately evaluate the pathology of each sample. We aimed to collect two samples from the tumor area, one histopathologically normal sample adjacent to the tumor (median 3 mm, range 0–12 mm) and one histopathologically normal sample distant from the tumor (median 24 mm, range 6–42 mm). If the prostate slice had two cancer lesions, we aimed to sample from both lesions.

One section from each sample was HES-stained and evaluated by histopathology to confirm the presence or lack of cancer tissue. The normal samples had only normal tissue, and the cancer samples had a median of 90% cancer area (range 10–100%). The samples in this study were selected from a bigger pool of samples selected for other spatial multi-omics analyses. Since this study uses bulk DNA methylation, we selected samples with less tissue heterogeneity in terms of differences in epithelial cell content, stroma content and inflammation were selected to reduce the confounding effect of tissue composition in analysis. At the same time, we ensured that the sample selection was representative of the bigger cohort in terms of similar EAU risk scores, and similar number of samples and patients with recurrence and metastasis.

DNA and RNA were isolated using the QIAGEN AllPrep DNA/RNA/miRNA kit (QIAGEN, Hilden, Germany) following the manufacturers protocol. The isolated DNA was bisulfite converted using the EZ DNA Methylation kit (Zymo Research, Orange, USA), and the converted DNA was analyzed for methylation using the Illumina HumanMethylationEPIC BeadChip following the manufacturers protocol.

### Methylation data preprocessing

The methylation data were preprocessed and normalized using the *minfi* package in R [14] and normalized using functional normalization (funnorm function). Probes with < 3 beads and a detection *p* value > 0.05 were removed. Probes with a SNPs less than 5 bp from the CpG were also removed to reduce confounding due to genetic variation, as described by Zhou et al. [15]. We used the Illumina annotation for identifying the CpGs relation to genes and CpG islands.

### Differential methylation analysis

For differential CpG analysis, we used a linear mixed model. This type of linear model accounts for the fact that some samples originate from the same patients, thereby enhancing the statistical power of the analysis in study designs that include multiple samples per patient. We used the *limma* package [16] in R to test all samples from recurrence patients against all samples from non-recurrence patients, using patient origin as random effect. Similarly, we tested samples from metastasis patients against non-metastasis patients. Significant sites were identified using an FDR adjusted *p* value of < 0.05. Heatmaps were made, and clustering was performed using the R package *ComplexHeatmap*.

### Methylation heterogeneity

Prostate cancer is often described as heterogeneous, but there is no standardized method for measuring and comparing this heterogeneity. To address this, we used two different analyses: variability measured by the coefficient of variation (CV) and similarity measured by correlation. The CV is calculated as the standard deviation divided by the mean and was chosen because it measures variability in a way that is comparable across different means. Additionally, we used correlation analysis in addition because even if something has “low heterogeneity,” it does not necessarily imply similarity. Thus, we quantify both variation and similarity within and between patients.

For the interpatient variability, we used one random sample per patient and calculated one CV per CpG across all the samples. We iterated the random sampling ten times and used the mean CV. For intrapatient variability, we used all samples per patient and calculated one CV for each CpG. To compare the CV of the DMCs (Differentially methylated CpGs) with methylation of other CpGs, we randomly sampled 37 CpGs to compare with the recurrence DMCs and 20 CpGs to compare with the metastasis DMCs and calculated the intra- and interpatient CV just as with the DMCs. The random sampling was iterated ten times, and we used the mean CV. Intra- and interpatient heterogeneity were compared using the Wilcoxon rank-sum test.

To assess the similarity of the methylation values within each patient, we correlated (Spearman’s ρ) the methylation values of the differentially methylated CpGs within the sample types (normal and cancer) within each patient. In case of less than two samples within a tissue type in one patient, we have no correlation coefficient, and in case of more than two samples within a tissue type, we used two random samples. For the comparison of normal samples versus cancer samples, we correlated the mean methylation of the cancer samples within each patient and the mean methylation of the normal samples within each patient. To compare the correlation of the DMCs with the correlation of methylation values across the rest of the array, we randomly sampled 37 CpGs and correlated the samples within each patient as for the DMCs. We performed the random sampling 100 times and used the mean of the correlations across the 100 iterations.

### Gene expression

Gene expression data were generated for all 64 samples. Library prep was performed using the cDNA library SENSE mRNA-Seq Library Prep Kit V2 (Lexogen, Vienna, Austria) with 600 ng RNA as input. Single-read sequencing was performed using the NextSeq 500/550 High Output kit v2.5 (75 cycles) on an Illumina NextSeq 500 instrument. Produced FASTQ files were filtered and trimmed using fastp v0.20.0. The sequence alignment was performed using the STAR tool against the reference genome set GRCh38 release 92 (Ensembl). Subsequently, *featureCounts* [17] was used to extract gene counts from sequence reads according to the reference set.

Differential analysis of gene expression was performed using linear mixed models with patient origin as random effect with the R package *edgeR* [18].

### Chromatin state assessment

To explore how the CpGs are related to chromatin states in prostate cancer tissue, we used ChromHMM data from prostate cancer cell lines. ChromHMM is a software for learning and characterizing chromatin states to functionally annotate the genome by using multivariate Hidden Markov Model for identifying combinatorial patterns of histone marks obtained from ChIP-seq data [19]. ChromHMM segmentation data were downloaded from the ENCODE data portal [20] from two prostate cancer cell lines: PC-3 (Accession ENCSR103ZFL) and LNCaP (Accession ENCSR072ZGM). The genomes were annotated into 17 distinct chromatin states and simplified as follows: Promoters were defined as "TssA," "TssBiv," "TssFlnk," "TssFlnkD," and "TssFlnkU"; enhancers were defined as "EnhA1,""EnhA2,""EnhBiv,""EnhG1,""EnhG2," and "EnhWk"; polycomb repressor complex (PRC) was defined as "ReprPC" and "ReprPCWk"; and transcribed was defined as "Tx" and "TxWk." Heterochromatin ("Het") and quiescent regions (low signal; "Quies") were left unchanged. Functional regions were intersected with CpG positions using BEDTools v2.27.1 [21].

Enrichment of CpGs in a ChromHMM defined functional region was measured as the ratio between the frequencies of identified CpGs of a specific functional region over the frequency of CpGs from the Illumina HumanMethylationEPIC array found within the same functional region. P-values were obtained by hypergeometric testing with the Illumina EPIC array probes as background. Multiple testing was accounted for using Benjamini–Hochberg correction.

### Enrichment for transcription factor binding regions

Maps of direct transcription factor-DNA (TF-DNA) interactions were obtained from the UniBind database (https://unibind.uio.no) [22]. The UniBind TFBSs (transcription factor binding sites) represent high-confidence direct TF-DNA interactions determined experimentally through ChIP-seq and computationally through position weight matrices (PWMs) from JASPAR [23]. The predicted transcription factor binding sites (TFBSs) were derived from 4114 ChIP-seq experiments for 268 TFs across cell types and tissues and were predicted to have high PWM scores and be near ChIP-seq peaks. TFBSs were extended by ± 150 bp (then called transcription factor binding regions; TFBRs) and were intersected with CpG positions using BEDTools v2.27 [21]. Since UniBind maps of TF binding sites are often derived from several ChIP-seq experiments for each TF, we merged the TF binding sites for all ChIP-seq experiments for each TF. Enrichment of CpGs in TFBRs was imputed using hypergeometric testing (R function phyper) using the IlluminaMethylationEPIC CpGs as background. Multiple testing was accounted for using Benjamini–Hochberg correction.

### Validation

We used the Fraser dataset [24] (GSE83917) as an external validation cohort. We used a Cox LASSO model for validating the differentially methylated CpGs. Since the validation dataset was analyzed using the Illumina HumanMethylation450 array, we used only the overlapping probes between the EPIC and HM450 array for training and testing. A LASSO Cox model was trained using leave-one-out-cross-validation. The alpha penalty was set to 0.1, to keep most DMCs in the model. The model was trained using all samples but one, and we used the model to predict the outcome of the “left out” sample, and we did this for all samples. The final model consisted of the DMCs that were in the model more than 50% of the iterations, and the coefficient used was the mean of all nonzero coefficients. The cutoff value used for separating the methylation groups was the median of the risk score.

## Results

### Differentially methylated CpGs associated with recurrence and metastasis

We identified 37 differentially methylated CpGs (DMCs) between samples from non-recurrence and recurrence when analyzing both normal, normal adjacent, and cancer samples together (Fig. 1a) (recurrence DMCs, FDR < 0.05). Using the identified DMCs, the samples clustered together according to recurrence status regardless of being classified as a cancer sample, normal, or normal adjacent sample (Fig. 1a). The normal adjacent samples and the normal distant samples also exhibit similar methylation patterns according to recurrence status, despite being extracted up to 39 mm apart. A patient example illustrating the similarity of methylation across the tissue slice is shown in Fig. 1c.

We further compared all samples from patients that developed metastasis during follow-up and those without metastases and identified 20 differentially methylated CpGs (metastasis DMCs, FDR < 0.05). Using these differentially methylated CpGs, the samples cluster together according to metastatic status regardless of being classified as cancer, normal distant, and normal adjacent (Fig. 1b), just as detected according to recurrence status (Fig. 1a). The normal samples from one recurrence patient, P21, were inaccurately clustered together with the non-recurrence normal samples, while the samples clustered correctly according to metastasis status. The recurrence DMCs and metastasis DMCs had 23% (17 617) overlapping probes in the top 10% sites (ranked by *p* value) and 9% overlapping probes (666) in the top 1% CpGs (Supplementary Fig. 1).

In conclusion, the differentially methylated CpGs discovered in this study separated the samples according to both recurrence and metastatic status regardless of tissue type and tumor grade. We identified two different sets of CpGs associated with recurrence and metastasis.

### Methylation is consistent across normal and cancer samples

**Fig. 2:**
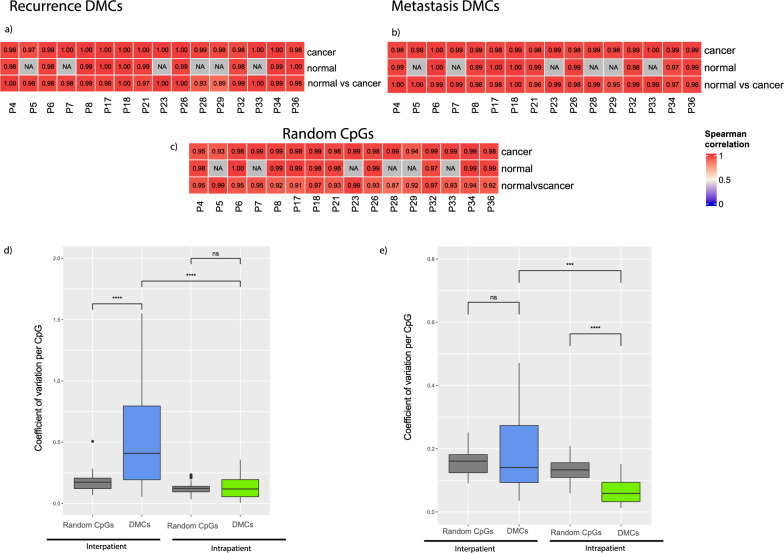
Intra- and interpatient heterogeneity of the recurrence-associated methylated CpGs (37 DMCs) and the metastasis-associated methylated probes (20 DMCs). **a**, **b** Intrapatient heterogeneity correlation analysis shows high correlation coefficients within tissue types in each patient both within the same tissue type and between cancer and normal tissue. NA values indicate that there was only one normal sample collected from the patient and correlation could, therefore, not be calculated. **c** Intrapatient correlation of randomly selected CpGs show high correlation for methylation values across the array. **d**, **e** The recurrence DMCs (**d**) had higher interpatient CV than a random selection representing the methylation of other CpGs. The metastasis DMCs (**e**) had a lower intrapatient CV than a subset of CpGs representing other CpGs. Ns = not significant, **p < 0.005, ***p < 0.0005, and ****p < 0.00005, Wilcoxon rank-sum

The methylation values of the recurrence and metastasis-associated DMCs in both cancer and normal tissue within the same patient were highly correlated, shown in Fig. 2a and b. The intrapatient Spearman correlation ranged from 0.89 to 1 in the recurrence DMCs, and from 0.95 to 1 in the metastasis DMCs, indicating that the methylation of these CpGs is independent of tissue morphology. The correlation within patients and tissue types were similarly high in randomly sampled CpGs from the whole array, except for the correlation between normal and cancer tissue, which was subtly but consistently lower for the random CpGs than the DMCs. The correlation values of the random CpGs suggest that the general similarity of methylation values across the prostate is high.

The variation between patients, given as the interpatient coefficient of variation (CV) was significantly higher for both recurrence DMCs (median CV 0.48) and metastasis DMCs (median CV 0.16) compared to the median intrapatient CV (recurrence DMCs 0.12 and metastasis DMCs 0.06). Compared to a random selection of 37 CpGs (median interpatient CV 0.17 and median intrapatient CV 0.12), the recurrence DMCs exhibited greater interpatient CV (*p* < 0.005), and a similar intrapatient CV (Fig. 2c). The metastasis DMCs had a similar intrapatient CV and lower interpatient CV than a random subset of 20 CpGs (interpatient CV 0.18 and intrapatient CV 0.14) in Fig. 2d.

Considering both the correlation analysis and the coefficient of variation, we conclude that the differentially methylated CpGs demonstrate low intrapatient heterogeneity.

### The DMCs associated with recurrence are located in promoter regions

**Fig. 3:**
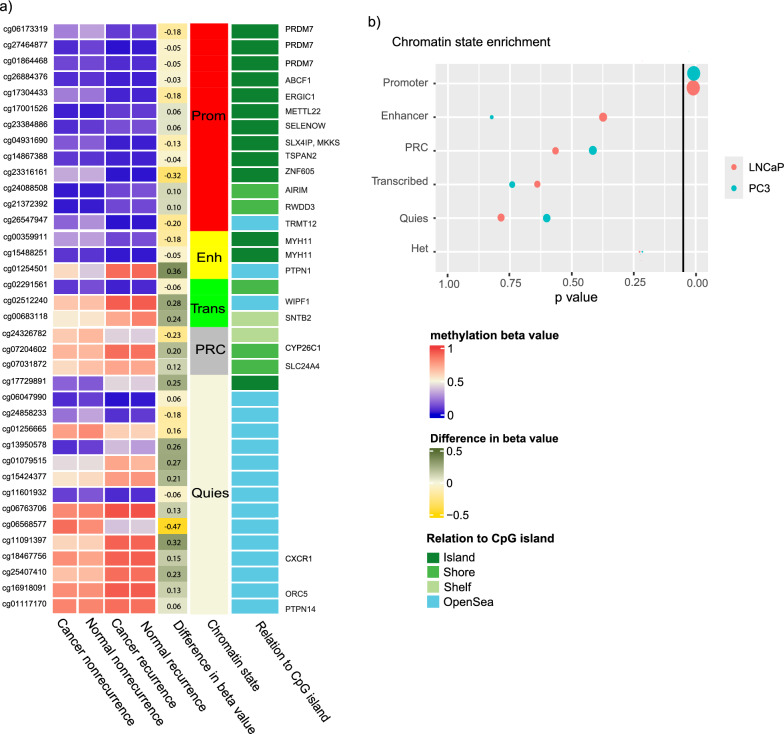
DNA methylation patterns and relation to CpG island and chromatin structure according to recurrence status. **a** Heatmap of the mean methylation beta values of recurrence and non-recurrence patients for the recurrence DMCs. Mean difference in beta value between recurrence and non-recurrence, chromatin state, and the relation to a CpG island is annotated. **b** The recurrence DMCs are enriched in promoter regions in prostate cancer cell lines PC-3 and LNCaP (adjusted p-value = 0.05), Prom = promoter, enh = enhancer, trans = transcribed, PRC = polycomb repressive complex, and quies = quiescent (low signal) state

We investigated the location of the DMCs both in context of chromatin structure and CpG islands to explore the potential biological associations of the DMCs (Fig. 3). We used ChromHMM datasets of prostate cancer cell lines (PC-3 and LNCaP) to assess the enrichment of the DMCs in chromatin states. The recurrence-associated DMCs were enriched in promoter regions, as defined by chromatin state (adjusted *p* value < 0.05), and 13 of the 14 promoter-CpGs were annotated to CpG islands. The metastasis-associated DMCs had no significant associations to chromatin states.

The expression of the 37 recurrence and 20 metastasis DMCs could be mapped to specific genes, 19 and 15, respectively, using Illumina CpG annotations. The expression of the genes annotated to the DMCs was not differentially expressed when comparing recurrence status, and the methylation and gene expression was not correlated (Pearson’s R < 60), however when comparing normal and cancer tissue, 17 of 19 genes were differentially expressed (*p* < 0.05, Supplementary Table 1). A similar pattern was observed comparing metastasis status; the genes annotated to the metastasis DMCs were not differentially expressed when comparing metastasis and non-metastasis patient samples, and the methylation and gene expression values were not correlated, however comparing normal and cancer tissue, 13 of 15 genes were differentially expressed (*p* < 0.05, Supplementary Table 2).

The CpGs annotated to CpG islands and promoters exhibit stable yet minor differences in beta values, with most variations likely being too small to significantly impact gene expression. There is a larger difference in beta value in the CpGs in open sea, regions of DNA where there are no CpG islands; however, the regulatory effect of these areas is harder to assess. In conclusion, both in the recurrence DMCs and metastasis DMCs, the DNA methylation pattern does not reflect gene expression, but we see a difference in gene expression between cancer and normal tissue.

### Recurrence-associated DMCs are enriched in transcription factor binding regions

**Fig. 4:**
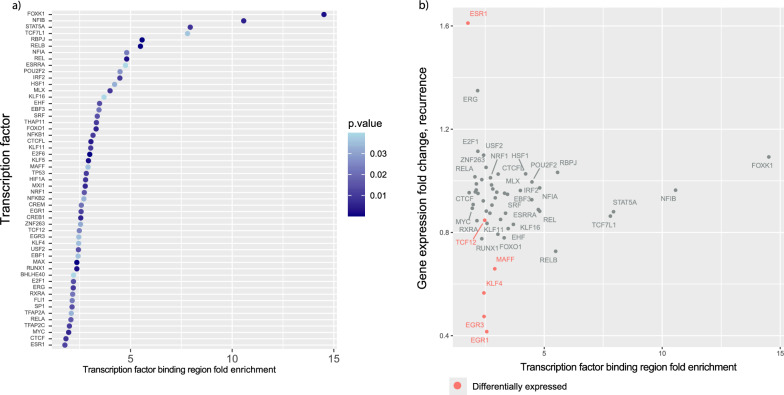
Transcription factors with enriched binding regions in the recurrence DMCs. **a** The significantly enriched transcription factor regions in the recurrence DMCs show a high enrichment for the transcription factors FOXK1, NFIB, STAT5A, and TCF7L1. **b** The significantly enriched transcription factors’ fold enrichment value in relation to the gene expression fold change. The fold change is calculated by comparing recurrence and non-recurrence samples. The transcription factors in red are differentially expressed in recurrence (*p* < 0.05, linear mixed model)

Differential methylation can also affect transcription factor binding. Therefore, we assessed if the identified CpGs were enriched in transcription factor binding regions. The recurrence DMCs were enriched in the binding regions of 53 transcription factors (TFs) (BH < 0.05) (Fig. 4a). The metastasis DMCs were not enriched in any transcription factor binding regions.

We then assessed the gene expression of these TFs using gene expression measurements from the same tissue samples as the methylation data (*n* = 64). *ESR1* was upregulated in recurrence patients, while *EGR3* and *KLF4*, as well as *MAFF*, *TCF12,* and *EGR1,* were downregulated. The CpGs were highly enriched in binding regions of *FOXK1*, *NFIB*, STAT5A, and *TCF7L1*, but these TFs showed no or minimal difference in expression fold change.

### A subset of the DMCs cannot identify groups with different risk of recurrence

The identified CpG sites show promise as tissue-based biomarkers because of their low intrapatient heterogeneity and high interpatient variation. We could not find open datasets for validation of recurrence outcomes in both cancer and normal samples using the same methylation array. Consequently, the full validation of the biomarker potential of the DMCs remains incomplete. Nevertheless, we conducted a partial validation using a dataset by Fraser et al. [24], that assessed methylation using the Illumina HM450 array and which included 22 of 37 of the recurrence DMCs.

**Fig. 5:**
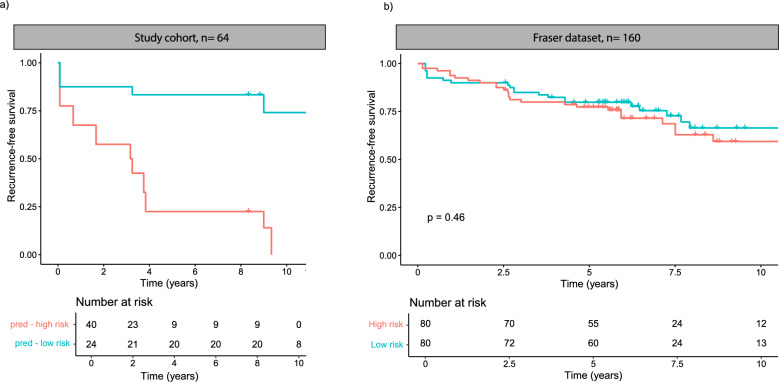
Kaplan–Meier plots showing the recurrence-free survival of the predicted methylation risk groups. There is a separation of a high- and low-risk group in our study cohort (**a**). In the validation dataset, there is no separation between the methylation risk groups (**b**)

Using a Cox LASSO model and selecting a subset of 22 of 37 of the recurrence DMCs, we separated the samples into two groups; the methylation high-risk group and methylation low-risk group, with a higher and lower risk of recurrence, respectively (Fig. 5a). The methylation risk groups were not predictive of recurrence in the Fraser dataset (*p* = 0.46).

## Discussion

We identified methylation aberrations in normal as well as cancer tissue from radical prostatectomy that were associated with recurrence and metastasis development after prostatectomy. These aberrations were present in the tissue at the time of surgery and in many patients several years before the tumor recurred or metastasized. The differences in methylation were enriched in promoter regions and transcription factor binding regions, regulatory areas of the genome, but were not directly associated to differences in gene expression. Since we observed methylation patterns that were not evident in gene expression, it shows the extra value of studying DNA methylation for biomarker development and discovery. This study also highlights the additional information that can be gained from studying tissue heterogeneity in multiple samples per patient, covering different tissue types and cancer grades.

The identified differential methylation patterns were present in normal tissue both adjacent to (< 1 mm) and on the other side of the prostate up to 4 cm away from the tumor. This provides further evidence for the epigenetic field effect in prostate cancer, which has been described both adjacent to and “far away” from the tumor [25], but not as far as in this study, and in fewer CpGs, using targeted methods instead of methylome-wide arrays. However, the field effect is sometimes found sporadically and not present in all samples or patients [26, 27], and may also be dependent on distance to the tumor [28], depending on the assessed CpGs. The long-distance methylation aberrations found in our study suggest that the field effect is present in the whole prostate, or that the CpGs we identified may not reflect the field effect but may indicate an individuals’ increased risk of developing aggressive and recurrent prostate cancer.

Our study design using multiple samples per patient including both normal and tumor tissue has enabled identification of DNA methylation sites that are associated with recurrence and metastasis risk and that have low intrapatient heterogeneity. A challenge for prostate cancer biomarker development using both DNA methylation and gene expression has been the high tissue heterogeneity. It is challenging to assess cancer risk and severity based on one cancer sample only as this sample may not represent the entire tumor or its most aggressive parts [29]. However, the intrapatient correlation of both the DMCs and random CpGs found in our study, suggest that the methylation values are similar across tissue types and sampling locations. The previous smaller studies on DNA methylation in prostate cancer targeting only a few specific CpGs have shown higher interpatient than intrapatient variation, consistent with our findings [30, 31]. Similarly, transcriptomic analyses have linked genes with low intrapatient heterogeneity to recurrence when analyzing several samples per patient [32]. While some CpGs, especially those related to cancer development, may exhibit high heterogeneity, CpGs with low variability across tissue types would be more robust as tissue-based biomarkers. Especially since the methylations patterns in our study were present regardless of tissue type. This illustrates the biomarker potential of our CpGs with low intrapatient and high interpatient heterogeneity to be useful for clinical future implementation.

DNA methylation can influence gene expression by directly affecting gene transcription, as well as by altering chromatin structure and transcription factor binding. Our study found that recurrence-associated differentially methylated CpGs (DMCs) were enriched in promoter regions. The histone mark H3K4me2, which indicates promoter state, has been independently linked to a higher risk of biochemical recurrence [33]. In addition, methylation differences in promoter regions suggest that these methylation differences likely play a role in gene regulation. However, when assessing the expression levels of methylated genes, we observe no differences comparing recurrence and non-recurrence patients nor metastasis and non-metastasis patients. However, there were differences in expression in normal and cancer tissue. This may have several possible explanations. Firstly, the observed differences in beta values may not have been substantial enough to impact gene expression, particularly for CpGs in promoter regions, which showed smaller beta value differences compared to CpGs in open sea regions. Open sea regions have more complex relationships with gene expression, making it challenging to determine if the methylation changes have specific biological effects. Secondly, the observed differences in methylation may reflect the cells’ potential for altered gene expression during subsequent stages of cancer progression. Thirdly, the genes may be regulated by other mechanisms than promoter methylation. Nonetheless, chromatin states alone do not influence gene expression without the involvement of transcription factor binding.

The differentially methylated CpGs were enriched in binding regions for several transcription factors. Methylation in transcription factor TF binding regions may enhance the binding of the transcription factor in that region, making it more likely for the TFs to initiate expression of downstream genes [34, 35]. In addition, binding of transcription factors may, in turn, affect methylation [36]. Therefore, assessing both the expression of the transcription factors and the target genes can be informative. However, given the vast number of potential targets for each TF, prostate-specific TF–target gene interaction data are essential to accurately interpret these regulatory relationships. In patients with recurrence, there was an increased expression of ERG and ESR1, which is linked to aggressive prostate cancer [37, 38]. Conversely, there was a decreased expression of the tumor suppressor genes EGR3, TCF12, and KLF4 [39–41]. EGR1, which may promote aggressiveness in prostate tumors, but appears protective against tumor progression in other cancer types, also showed lower expression in recurrence patients [42, 43]. The recurrence-associated CpGs were enriched in transcription factor binding regions of FOXK1 and NFIB. Although these transcription factors did not show differential expression at the time of prostatectomy, they may become relevant at later stages of disease, as both are associated with aggressive features in prostate cancer [44, 45]. Together, these methylation changes and differences in transcription factor expression may play a role in the transition from normal prostate cell biology to aggressive cancer enabling higher sensitivity of TF binding associated with aggressiveness.

The differential methylation linked to metastasis primarily involved a loss of methylation, a phenomenon previously described in aggressive and metastasizing prostate cancer and thought to occur later in disease progression [46]. Interestingly, we find these aberrations in normal prostate tissue years before the patients are diagnosed with metastases which can be a marker to describe a patient’s vulnerability to recur or metastasize. Given the small sample size, further investigation in larger study cohorts is warranted.

We were unable to validate our findings in an independent dataset due to the lack of available methylation datasets that included all differentially methylated CpGs (DMCs) and clinical recurrence and metastasis follow-up data. To determine the biomarker potential of these CpGs, further validation in a dataset encompassing all DMCs is necessary. However, we attempted to validate a subset of the DMCs (22 of 37) that overlapped with the detected probes in an external dataset which used an older Illumina methylation array. The prediction model we constructed from our methylation data was not predictive in the Fraser dataset [24]. Given the differences in cancer aggressiveness and grade—with the Fraser dataset containing mostly low-risk patients and our dataset containing intermediate- and high-risk patients—it is challenging to determine whether the failed validation was due to differences in the datasets or the actual methylation of the CpGs.

## Conclusion

In conclusion, our study identified differentially methylated CpGs linked to both recurrence and metastasis of prostate cancer. These CpGs exhibited consistent methylation patterns regardless of tissue type and sampling location, making them viable candidates for clinical biomarkers in normal-appearing tissue. Our findings lay the groundwork for further validation and potential clinical application.

## Supplementary Information

Additional file1 (DOCX 66 KB)

Additional file2 (TXT 2 KB)

Additional file3 (TXT 2 KB)

## Data Availability

The data generated and analyzed in this study includes sensitive information, and its management must comply with the General Data Protection Regulation (GDPR), Norwegian law, and the specific patient consent and ethical approval. Consequently, the data is legally subjected to restricted access. Raw and processed transcriptomics and DNA methylation data have been deposited at Federated European Genome-Phenome Archive (FEGA) Norway and are findable on the EGA portal (ega-archive.org) under the study ID EGAS50000000413. The transcriptomics and DNA methylation data are deposited as separate datasets with the accession numbers EGAD50000000604 and EGAD50000000605, respectively. Data access can be requested through the EGA portal, where any data request will be processed through a data access committee at NTNU. Access will only be granted after the following steps have been achieved; 1. the data requester and the intended use of the data must comply with GDPR regulation, Norwegian law, and the specific patient consent, 2. data sharing with the specific data requester must be approved by the regional ethical committee (REC) in Norway, 3. the Data Protection Impact Assessment (DPIA) may require revision and 4. there must be a signed data transfer agreement between the institution of the data requester and NTNU. Depending on the intended use of the data, the data requester can also be required to establish a collaboration agreement with NTNU prior to data sharing.
