# Supplementary material for: DNA methylation in normal-appearing tissue associated with prostate cancer recurrence and metastasis
Source: Clin Epigenetics. 2025 Jul 21;17:127. doi: 10.1186/s13148-025-01932-x (PMC12281782; doi:10.1186/s13148-025-01932-x)
Supplement: Supplementary file 1 — Additional file1 (DOCX 66 KB) [file 13148_2025_1932_MOESM1_ESM.docx]

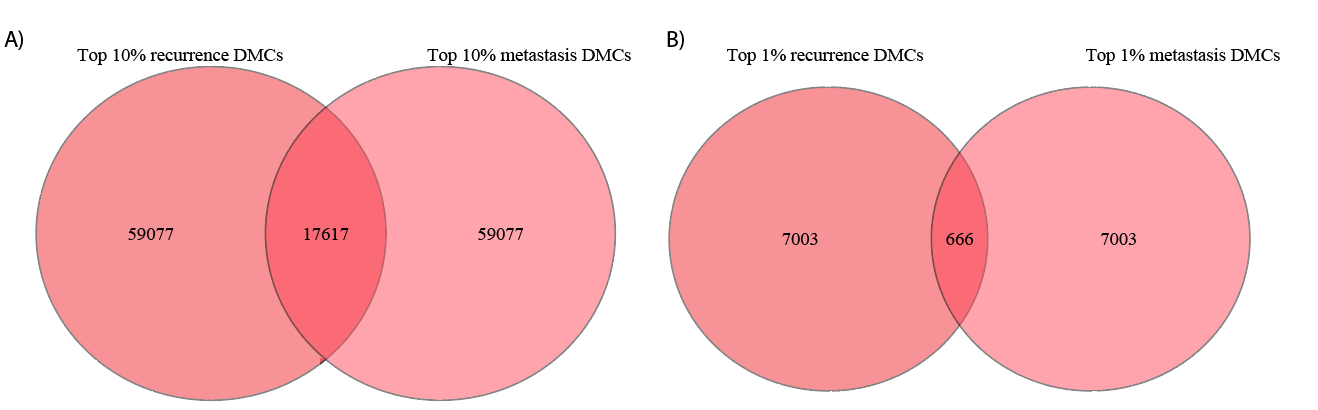


Supplementary Figure 1: Visual representation of the overlap between the recurrence DMC and the metastasis DMCs in A) the top 10% CpGs ranked by p-value and B) the top 1% CpGs ranked by p-value.
